# A Sleep and Circadian Biomarker‐Based Predictive Model for Differentiating Unipolar and Bipolar Depression

**DOI:** 10.1155/da/5556144

**Published:** 2026-09-15

**Authors:** Jeanne Leseur, Pierre A. Geoffroy, Anne Perozziello, Delfine Abderrahmane, Héloïse Rach, Michel Lejoyeux, Marie Pia d’Ortho, Justine Frija, Julia Maruani

**Affiliations:** ^1^ Department of Psychiatry and Addictology, AP-HP, GHU Paris Nord, DMU Neurosciences, Bichat–Claude Bernard Hospital, Paris F-75018, France, aphp.fr; ^2^ NeuroDiderot, Inserm, Université Paris Cité, Paris F-75019, France, u-paris.fr; ^3^ Department of Psychiatry and Neurosciences, GHU Paris Nord, Bichat–Claude Bernard Hospital, Paris F-75018, France; ^4^ Faculty of Medicine, Aix-Marseille Université, Marseille F-13007, France, univmed.fr; ^5^ Department of Clinical Physiology, Functional Explorations and Sleep Center, AP-HP, Bichat–Claude Bernard Hospital, Paris F-75018, France, aphp.fr

**Keywords:** actigraphy, bipolar disorder, circadian rhythm, depressive disorder, polysomnography, psychomotor disorders, sleep wake disorder

## Abstract

**Background/Study Objectives:**

Differentiating unipolar depression (UDD) from bipolar depression (BDD) remains a major clinical challenge with important treatment implications. Sleep‐related markers, both subjective (such as hypersomnia in BDD and insomnia in UDD) and objective (actigraphy, polysomnography [PSG]), show promise, yet no study has integrated these approaches into a single predictive model.

**Methods:**

Patients with DSM‐5‐TR‐defined UDD or BDD in a depressive episode underwent clinical, questionnaire, actigraphy, and PSG assessments. Discriminating variables were entered into a backward stepwise logistic regression (BSLR) to derive the optimal classification model.

**Results:**

The study drew on 159 patients: 43 with BDD (42 actigraphy, 20 PSG) and 116 with UDD (93 actigraphy, 44 PSG). Compared to BDD, patients with UDD reported poorer sleep quality (Pittsburgh sleep quality index [PSQI]), more severe insomnia (insomnia severity index [ISI]), and lower sleep efficiency (SE, actigraphy). Patients with BDD showed longer total rest time per 24 h, lower average activity during the least active 5‐h period (L5) and the 10 most active hours (M10), and higher N2% and total NREM sleep (PSG). Six variables were retained in the final BSLR model, explaining 49.4% of the variance. The most discriminative were higher PSQI/ISI and lower actigraphic SE in UDD, versus longer rest time, lower L5 activity, and higher N2% in BDD. The model showed excellent discriminative ability (AUC = 0.926, sensitivity = 0.882, specificity = 0.895, Youden’s index = 0.777), with strong predictive values (positive predictive value [PPV] = 88.3%, negative predictive value [NPV] = 89.5%).

**Conclusion:**

Integrating subjective, actigraphic, and polysomnographic markers provides a promising, noninvasive approach to differentiate UDD from BDD.

## 1. Introduction

Mood disorders encompass a group of highly prevalent and disabling psychiatric conditions, affecting up to 20% of the population and ranking among the leading causes of disability worldwide [1]. Within this spectrum, a major diagnostic challenge lies in distinguishing unipolar depression (UDD) from bipolar depression (BDD), particularly during major depressive episodes (MDEs), which often represent the initial clinical presentation of bipolar disorder (BD). This diagnostic ambiguity contributes to a significant delay, estimated at 8–10 years, before appropriate treatment is initiated [2]. It also increases the likelihood of inappropriate interventions, such as prescribing antidepressants to patients with undiagnosed BD. Such mismanagement can worsen the clinical course, increase morbidity, and elevate the suicide risk [3, 4]. In this context, the development of predictive tools to aid early and accurate diagnostic differentiation between patients with UDD and BDD is critical for improving treatment selection and clinical prognosis.

In recent years, increasing attention has been directed toward identifying biomarkers that could support this diagnostic distinction. Several biological domains have been explored, including inflammatory markers, neuroimaging findings, genetic vulnerability, and neurocognitive profiles [5–7]. While these approaches have shown some differences between BDD and UDD, their findings often rely on costly or invasive assessments and still require replication before their diagnostic robustness can be firmly established. In the present study, we focus on sleep and circadian rhythm disturbances because they represent a particularly relevant area of investigation. Sleep disturbances are closely and bidirectionally linked to mood regulation, are highly prevalent throughout the course of mood disorders, including during euthymic phases, and are associated with illness severity [8–11]. Moreover, emerging evidence suggests that specific sleep and circadian features may differ between BDD and UDD [12–17], making them promising candidates for improving diagnostic differentiation. Importantly, a wide range of complementary assessment tools exists for these measures, offering practical options for their use in clinical settings.

Together, these investigations have contributed to identifying distinct sleep profiles and refining the differential characterization of these disorders.

Clinically, hypersomnolence complaints appear to be more common in BDD patients [16, 18, 19], whereas insomnia complaints, especially sleep‐onset insomnia, are more frequently observed in UDD [17–19]. However, symptoms frequently overlap, and up to a third of BDD patients report both insomnia and hypersomnolence complaints during depressive episodes [9], limiting the specificity of subjective measures alone.

To further explore sleep‐related mechanisms, objective assessments using polysomnography (PSG) have been extensively employed. PSG is the gold standard for studying sleep macrostructure and provides information on sleep continuity, depth, and sleep stage distribution. Despite its advantages, findings from studies comparing patients with BDD and UDD using PSG remain inconsistent. Some have reported longer REM sleep latency [14], increased REM fragmentation [20], prolonged sleep latency [12], and fewer nocturnal awakenings [13] in BDD compared to those in UDD. Other studies have found no significant differences between the two groups [21–23]. These discrepancies may stem from methodological heterogeneity and from the limited statistical power inherent in PSG‐based studies due to their high cost and logistical constraints [24].

In light of these methodological and practical limitations, actigraphy has emerged as a valuable complementary approach. Validated against PSG, actigraphy enables the assessment of sleep–wake patterns over extended periods and in naturalistic settings, thus offering improved ecological validity and a larger sampling potential [25, 26]. Importantly, certain sleep parameters have been examined across both PSG and actigraphy‐based studies. Emerging evidence suggests that BDD is often associated with longer total sleep time (TST) [13, 14], while those with UDD consistently show lower SE [27]. These patterns are in line with the subjective differences reported between the two disorders. A recent meta‐analysis conducted within our department reinforced these trends [15], although stage‐specific differences could not be evaluated due to the limited statistical power in the original datasets.

In addition to sleep‐related measures, circadian rhythm disruptions represent another key dimension relevant to differential diagnosis. Indeed, they have already been used to distinguish patients with BD who were in the euthymic phase from healthy controls [28], as well as from slowed‐down and accelerated forms of depression [29].

Given the strong links between mood disorders and circadian dysregulation [30, 31], such parameters are of particular relevance in distinguishing between BDD and UDD. However, few studies have identified consistent circadian rhythm differences between the two disorders, with the exception of a study by Robillard et al. [32] reporting a delayed sleep phase in BDD relative to UDD, in younger populations. Alongside circadian measures, increasing attention has also been directed toward psychomotor activity, which reflects daily activity–rest rhythms and is increasingly regarded as a behavioral manifestation of circadian functioning. Several actigraphy‐based studies have reported reduced motor activity in BDD compared with that in UDD [19, 33], consistent with clinical observations [34].

Taken together, sleep architecture, circadian rhythms, and psychomotor activity each provides complementary information relevant to mood disorder characterization. However, most previous studies have examined these dimensions separately using either subjective questionnaires, actigraphy, or PSG‐based approaches. As a result, findings remain fragmented across methodological levels, and it remains unclear which combination of parameters provides the greatest discriminative value in clinical populations. Importantly, although individual sleep and circadian markers may show group‐level differences, none have demonstrated sufficient accuracy to be used in isolation as a clinical decision tool.

Rather than identifying a single biomarker, the current challenge therefore lies in determining whether an integrative and multimodal approach combining subjective, objective, sleep, and circadian measures can improve the differentiation between BDD and UDD. To date, no validated diagnostic model has systematically integrated these complementary dimensions within a unified framework for routine clinical use. Accordingly, the aim of the present study is to evaluate how their integration may contribute to improving the differential diagnosis between BDD and UDD.

In this context, the aim of our study was to develop a predictive model for use in routine clinical settings based on a noninvasive, longitudinal assessment of subjective and/or objective sleep and circadian markers. Such an approach could contribute meaningfully to the early identification of mood disorder subtypes and support the development of more individualized and effective treatment strategies.

## 2. Materials and Methods

### 2.1. Study Design

This is a comparative study of patients suffering from MDE with unipolar disorder (UD) and BD. Sleep and circadian markers were evaluated by subjective (self‐report questionnaires and hetero‐assessment) and objective (actigraphy for at least 14 days and PSG) measures.

These patients were drawn from the routine care cohort from the project Sompsy (“study of biomarkers of sleep and circadian rhythms in psychiatric disorders,” PI: PA Geoffroy) in the university psychiatric and addiction department of Bichat Claude Bernard Hospital. It has been ethically approved (Number CER‐2020‐56) by the “comité d’évaluation de l’éthique des projets de recherche biomédicale” (CEERB), Paris Nord.

### 2.2. Sociodemographic and Clinical Parameters

Patients from this clinical cohort suffer from MDE according to the DSM‐5‐TR [35] as made by a trained psychiatrist. For the present study, patients receiving routine care at a single medical center (Hospital Bichat–Claude Bernard) between July 2022 and June 2025 were considered. Patients were adults aged between 18 and 70 years old and were diagnosed with UDD or BDD according to the DSM‐5‐TR [35]. Patients with psychotic disorders, except those with psychotic symptoms related to the current depressive episode, were not considered.

As participants were drawn from a naturalistic care setting, pharmacological treatments were prescribed according to the usual clinical practice.

All patients received oral information and a written note explaining the cohort project. A note of nonopposition was kept in the patient medical record. All clinical data were collected and recorded in a database where all patients were assigned an anonymous number.

### 2.3. Measures in Sleep, Awake, Circadian, and Seasonality Rhythms

#### 2.3.1. Clinical Evaluation

The clinical evaluation by the trained psychiatrist and sleep physician, based on the criteria of the DSM‐5‐TR [35], systematically assessed sleep disorders such as insomnia, defined as a complaint of reduced quantity or quality of sleep, and hypersomnia, defined as an excessive amount of sleep.

#### 2.3.2. Self‐Report Assessment

As part of our routine clinical assessments [36], patients completed the French versions of a battery of validated sleep and circadian rhythm questionnaires, capturing complementary dimensions of sleep and sleep–wake functioning. Insomnia severity, sleep quality, and hypersomnolence were assessed using the insomnia severity index (ISI) [37], the Pittsburgh sleep quality index (PSQI) [38], and the hypersomnia severity index (HSI), respectively [39]. Daytime sleepiness was measured with the Epworth sleepiness scale (ESS) [40] and circadian typology with the morningness–eveningness questionnaire (MEQ) [41]. Anxiety symptoms were assessed using the generalized anxiety disorder‐7 (GAD‐7) [42].

#### 2.3.3. Hetero‐Assessment

The patients had a hetero‐assessment by the psychiatrist to evaluate the severity of the depression by using the Montgomery and Asberg scale (MADRS) [43].

#### 2.3.4. Actigraphy

In the context of routine care, some patients were assessed with actigraphy (MotionWatch8, camNtech Ltd., Cambridge, UK) worn on the wrist of their nondominant hand for at least 14 days [26], starting on the day they filled out the questionnaire. Actigraphy is a noninvasive, ambulatory accelerometer that provides objective information about sleep–wake rhythms, nonparametric circadian rhythm analysis (NPCRA), light exposure, and physical activity analysis. Recordings were taken in 60‐s epochs. At the end of the wear period, data were downloaded using the MotionWare 8 software (Version 1.3.17). Actigraphy parameters used in our study were: (1) TST reflects total time effectively spent asleep (in hours); (2) wake after sleep onset (WASO) represents the period of wake time in the sleep period (in hours); (3) sleep efficiency (SE) represents TST/time in bed (in percentage); (4) sleep‐onset latency (SOL) represents the difference between bedtime and sleep (in minutes); (5) rest per 24 h represents the percentage of epochs in a 24 h period which could be counted as sleep/rest if the whole period were analyzed as night time; (6) L5 onset represents the starting hour of the 5 less active hours; and (7) L5 average represents the activity amplitude of the 5 least active hours. The average activity gives an indication of how restful the rest (sleep) periods are: (8) M10 onset represents the starting hour of the 10 most active hours; (9) M10 average represents the 10 most active hours. The average activity gives an indication of the peak of the rhythm: how intense is the activity during the most active period. (10) Interdaily stability (IS) represents the degree of regularity of the activity–rest pattern. (11) Intradaily variability (IV) represents the degree of fragmentation of rest‐activity periods.

Actigraphy has been validated for studying sleep disorders and circadian rhythms as compared to the gold standard (PSG) by the American Academy of Sleep Medicine (AASM) [26], notably in BD [25]. It should be noted that the selection of markers analyzed is modeled after our previous research [19].

#### 2.3.5. PSG

In the context of routine care, some patients underwent one night of PSG using a standard system (V‐PSG; Morpheus by Micromed; Noxturnal by ResMed; and Alice 5, Philips‐Respironics, Suresnes, France). The recorded parameters included electroencephalogram (EEG) with six channels (F3A2, F4A1, C3A2, C4A1, O1A2, and O2A1); electrooculography; submental and tibial electromyography; electrocardiography; oronasal airflow measured using a thermocouple; respiratory effort measured using inductive plethysmography, with one thoracic and one abdominal belt; and arterial oxyhemoglobin saturation measured using pulse oximetry. Sleep medicine physicians manually scored the sleep stages in accordance with the clinical scoring guidelines established by the AASM [44]. The following sleep variables were computed for descriptive purposes: (1) TST (in minutes), (2) SE (in percentage), (3) SOL (in minutes), (4) REM latency, which is the time between sleep onset and the first occurrence of REM sleep (in minutes). (5) Proportion of TST spent in stage N1, in stage N2, and in stage N3: N1% TST, N2% TST, and N3% TST (percentage of TST). (6) Total NREM sleep time: was calculated as the sum of time spent in stages N1, N2, and N3. It corresponds to the overall duration of non‐REM sleep, which reflects a progressive slowing of brain activity from light sleep stages (N1 and N2) to deep sleep (N3) (in minutes). (7) Time spent in REM sleep: REM% TST (percentage of TST). TST and SE had been analyzed as candidate markers, given that they had previously been shown to differ between groups in other studies [13, 14, 19, 27]. All other markers had been examined in an exploratory manner, as they had been considered in prior research but had not consistently been found to differ [12, 14, 20, 27].

### 2.4. Statistical Analysis

In the first step, quantitative results are expressed as mean and standard deviation and categorical variables as percentage. The normality of the continuous variable distribution was assessed with the statistical test of normality (Shapiro–Wilk test *p* > 0.05) and we looked at the equality of variances between the two groups for the measured variable (Levene test *p* < 0.05). We compared the two groups using parametric (*t*‐student) or nonparametric tests (*U* Mann Whitney, Welch) in case of nonnormal distribution or in case of nonequality of the variances. Categorical variables were compared using the Chi2 test. This univariate analysis was conducted with an exploratory aim to draw on as many patients and potential discriminative variables as possible. The goal was to identify variables showing statistically significant differences (*p* < 0.05) between patients diagnosed with UDD and BDD.

In a second step, all variables that showed statistically significant differences between the groups (*p* < 0.05) were entered into a backward stepwise logistic regression (BSLR) model. The least statistically significant variables were sequentially removed at each step until the model with the best overall fit was obtained (with the method previously detailed in [45], based on [46]). The number of retained variables was therefore not predetermined.

For this multivariate analysis, a sample size calculation was performed a priori, requiring at least 20 individuals per variable.

This analysis aimed to identify the combination of these variables that best discriminates between individuals with UDD and those with BDD.

Before conducting the regression analyses, the absence of multicollinearity among the variables was verified. Odds ratios (ORs) and their 95% confidence intervals were estimated, and a *p*‐value < 0.05 was considered statistically significant. Medication classes (antidepressants and mood stabilizers) were not included as covariates in the BSLR model, given their strong association with diagnostic status.

To assess the discriminative ability of the selected predictors, receiver operating characteristic (ROC) curves and the corresponding area under the curve (AUC) were computed. Sensitivity and specificity values were determined based on these ROC curves, and positive predictive value (PPV) and negative predictive value (NPV) were also calculated. An optimal threshold for classifying patients diagnosed with UDD and BDD was identified using the Youden’s index (Youden’s *J* = sensitivity + specificity – 1). The significance of this global model was also evaluated, and its variance was assessed, including the *R*‐squared value.

Analyses were performed using JAMOVI software (2.3.26) [47]. For all tests, a value of *p* < 0.05 was considered significant.

## 3. Results

### 3.1. Sample Characteristics

One hundred and fifty‐nine patients with MDE were analyzed between July 2022 and June 2025. Among these patients, 43 had a diagnosis of BDD, while 116 had a diagnosis of UDD (see Table 1).

**Table 1:** Sociodemographic and clinical characteristics of patients with bipolar depression BDD (BDD, *n* = 43) and unipolar depression (UDD, *n* = 116).

Variables	Groups mean (SD) or *n* (%)		Comparison UDD vs. BDD	
	UD (*n* = 116)	BDD (*n* = 43)	Khi^2^, Mann–Whitney *U*, *t*‐student, Welch	*p*
Age (years)	39.50 (±13.32)	43.49 (±13.28)	−1,677	0.098
Gender distribution				
Female	73 (62.9%)	25 (58.1%)	0.305	0.581
Male	43 (37.1%)	18 (41.9%)		
Age of onset (years)	25.1 (±10.6)	23.0 (±9.62)	1572	0.430
Personal Hx of trauma				
Yes	61 (53%)	18 (42.9%)	1.28	0.258
No	54 (47%)	24 (57.1%)		
BMI (kg/m^2^)	26.7 (±7.19)	25.1 (±4.27)	1.68	0.095
Rating scales				
MADRS	25.03 (±7.75)	24.32 (±7.32)	1897	0.970
GAD‐7	12.62 (±0.476)	12.76 (±0.785)	2228	0.801
Sleep symptoms of the current major depressive episode				
Insomnia				
Yes	98 (84.5%)	31 (73.8%)	2.34	0.126
No	18 (15.5%)	11 (26.2%)		
Hypersomnia				
Yes	22 (19.3%)	14 (33.3%)	3.41	0.065
No	92 (80.7%)	28 (66.7%)		

Abbreviations: BMI, body mass index; GAD‐7, generalized anxiety disorder‐7; Hx, history; MADRS, Montgomery‐Åsberg depression rating scale.

No significant differences were found between the groups regarding age, gender, body mass index, family history of MDE, personal history of traumatism, and the severity of depression and anxiety.

Regarding clinical sleep symptoms, no significant differences were found between the two groups for insomnia, while hypersomnia showed a nonsignificant trend toward higher prevalence in the BDD group (*p* = 0.065).

### 3.2. Sleep and Circadian Rhythms Assessment

Significant differences were observed between the UDD and BDD groups in various subjective sleep quality measures and insomnia severity (Table 2). The UDD group reported poorer sleep quality (PSQI, *p* = 0.001), including lower subjective quality (PSQI subjective quality, *p* = 0.010), longer SOL (PSQI sleep latency, *p* = 0.018), lower SE (PSQI SE, *p* < 0.001) and more sleep disturbance (PSQI sleep disturbance, *p* < 0.001) compared to the BDD group. Also, UDD reports more severe insomnia symptoms (ISI, *p* = 0.026). In contrast, the BDD group reported longer sleep duration (PSQI sleep duration, *p* = 0.014) compared to the UDD group.

**Table 2:** Comparison of patients diagnosed with BDD (*n* = 43) and UDD (*n* = 116) regarding subjective sleep and circadian rhythms markers assessed with questionnaires.

Variables	Groups mean (SD) or *n* (%)		Comparison UDD vs. BDD	
	UDD (*n* = 116)	BDD (*n* = 43)	*t*‐Student, Mann–Whitney *U*	*p*
ISI	18.98 (±4.87)	16.60 (±5.78)	1662	**0.026**
PSQI	12.83 (±3.83)	10.61 (±3.51)	3.244	**0.001**
Subjective quality	2.36 (±0.68)	1.93 (±0.95)	1800	**0.010**
Sleep latency	2.02 (±0.94)	1.62 (±0.96)	1811	**0.018**
Sleep duration	1.11 (±1.20)	0.64 (±0.98)	2.499	**0.014**
Sleep efficiency	1.56 (±1.20)	0.83 (±1.08)	1551	**<0.001**
Sleep disturbance	1.91 (±0.67)	1.50 (±0.60)	1635	**<0.001**
Sleep promoting medication	1.98 (±1.30)	2.38 (±1.13)	−1.881	0.063
Daytime dysfunction	1.88 (±0.78)	1.73 (±0.78)	2096	0.333
ESS	9.17 (±5.32)	8.65 (±5.35)	2296	0.541
MEQ	46.24 (±11.17)	45.77 (±13.51)	0.213	0.832
HSI	18.43 (±5.33)	17.38 (±5.86)	0.964	0.337

*Note*: Bold values indicate *p* < 0.05.

Abbreviations: ESS, Epworth sleepiness scale; HSI, hypersomnia severity index; ISI, insomnia severity index; MEQ, morningness–eveningness questionnaire; PSQI, Pittsburgh sleep quality index.

There were no significant differences in terms of the use of sleep‐promoting medication and daytime dysfunction in PSQI, daytime sleepiness assessed by ESS, chronotype by MEQ, and hypersomnia severity by HSI.

### 3.3. Actigraphy

As reported in Table 3, significant differences were observed in objective sleep and circadian rhythm measures between patients with UDD and BDD. In terms of sleep parameters, the UDD group showed lower SE compared to the BDD group (80.70% vs. 83.62%, *p* = 0.026). Regarding circadian measures, the BDD group exhibited a higher percentage of rest over 24 h (49.10% vs. 44.15%, *p* = 0.017), a lower L5 average (784.52 vs. 1105.48, *p* = 0.004), and a lower M10 average (10028.09 vs. 12308.83, *p* = 0.024).

**Table 3:** Sleep and circadian characteristics of patients with BDD (*n* = 42) and UDD (*n* = 93) assessed by actigraphy.

Variables	Groups mean (SD) or *n* (%)		Comparison UDD vs. BDD	
	UDD (*n* = 93)	BDD (*n* = 42)	*t*‐Student, Mann–Whitney *U*, Welch	*p*
TST (hh:mm:ss)	7:05:05 (±1:12:12)	7:27:57 (±1:26:56)	−1.488	0.141
WASO (hh:mm:ss)	1:23:27 (±0:35:27)	1:10:51 (±0:30:25)	1595	0.90
SE (%)	80.703 (±7.06)	83.619 (±6.807)	−2.247	**0.026**
SOL (hh:mm:ss)	0:13:36 (±0:12:18)	0:13:30 (±0:23:42)	0.036	0.971
Rest over 24 h (%)	44.147 (±11.079)	49.102 (±11.052)	−2.407	**0.017**
L5 onset (hh:mm:ss)	3:25:08 (±5:24:50)	2:47:08 (±5:35:53)	1536	0.076
L5 average	1105.478 (±848.402)	784.524 (±822.565)	1295	**0.004**
M10 onset (hh:mm:ss)	10:17:20 (±2:17:21)	10:10:00 (±1:48:24)	1791	0.626
M10 average	12308.833 (±5006.364)	10028.095 (±4495.531)	1427	**0.024**
Interdaily stability (IS)	0.375 (±0.119)	0.386 (±0.129)	−0.492	0.623
Intradaily variability (IV)	0.871 (±0.193)	0.906 (±0.237)	1765.5	0.545

*Note*: Bold values indicate *p* < 0.05.

Abbreviations: L5 average, average activity of the 5 least active hours; L5 onset, onset of the least active 5‐h period; M10 average, average activity of the 10 most active hours; M10 onset, onset of the most active 10‐h period; SE, sleep efficiency; SOL, sleep‐onset latency; TST, total sleep time; WASO, wake after sleep onset.

No significant group differences were found for other sleep parameters (TST, WASO, and SOL) or circadian variables (L5 onset, M10 onset, IS, and IV).

### 3.4. PSG

As shown in Table 4, significant differences in polysomnographic sleep parameters were observed between the two groups. Patients with BDD exhibited a higher percentage of N2 sleep during the night compared to those with UDD (56.56% vs. 48.30%, *p* = 0.005), but a lower percentage of N3 sleep (19.38% vs. 25.64%, *p* = 0.029). In addition, BDD patients displayed a longer duration of NREM sleep (05:49:38 vs. 04:59:16, *p* = 0.013).

**Table 4:** Sleep characteristics of patients with BDD (*n* = 20) and UDD (*n* = 44) assessed by polysomnography.

Variables	Groups mean (SD) or *n* (%)		Comparison UDD vs. BDD	
	UDD (*n* = 44)	BDD (*n* = 20)	*t*‐Student, Mann–Whitney *U*, Welch	*p*
TST (hh:mm:ss)	6:36:06 (±1:34:00)	7:35:30 (±1:21:36)	−1.920	0.060
WASO (hh:mm:ss)	0:52:43 (±1:03:26)	0:59:00 (±1:02:44)	91	0.647
SE (%)	79.1 (±16.2)	85.3 (±14.6)	133	0.582
SOL (hh:mm:ss)	00:33:20 (±00:48:28)	00:25:02 (±00:43:42)	158	0.937
N1 TST (%)	8.718 (±7.725)	7.585 (±6.319)	0.574	0.568
N2 TST (%)	48.298 (±10.966)	56.560 (±9.151)	−2.934	**0.005**
N3 TST (%)	25.641 (±11.644)	19.380 (±6.673)	2.237	**0.029**
Duration of NREM (N1 + N2 + N3) (hh:mm:ss)	04:59:16 (±01:16:32)	05:49:38 (±01:04:44)	−2.554	**0.013**
REM TST (%)	17.436 (±8.523)	16.375 (±8.198)	0.467	0.642
REM latency (min)	140.82 (±83.45)	208.98 (±167.54)	−1.547	0.139

*Note*: Bold values indicate *p* < 0.05.

Abbreviations: NREM, nonrapid eye movement; REM, rapid eye movement; SE, sleep efficiency; SOL, sleep‐onset latency; TST, total sleep time; WASO, wake after sleep onset.

A trend toward a longer TST was also observed in the BDD group compared to the UDD group (07:35:30 vs. 06:36:06, *p* = 0.060), although this difference did not reach statistical significance.

No significant group differences were found for WASO, SE, SOL, REM TST%, or LREM.

### 3.5. Multivariate Analysis to Identify Independent Factors Predicting the UDD and BDD Group

We next applied BSLR. A total of 36 patients were included (19 with UDD and 17 with BDD); no evidence of multicollinearity was found among the variables (Table S6). After entering the variables that showed significant differences between the UDD and BDD groups in the univariate analysis, a combination of six variables was retained: patients diagnosed with UDD showed more severe insomnia symptoms (ISI), lower sleep quality (PSQI), and efficiency (actigraphy) compared to BDD who instead exhibited lower activity during L5 average, more rest over 24 h, and a higher N2% TST.

**Figure 1:**
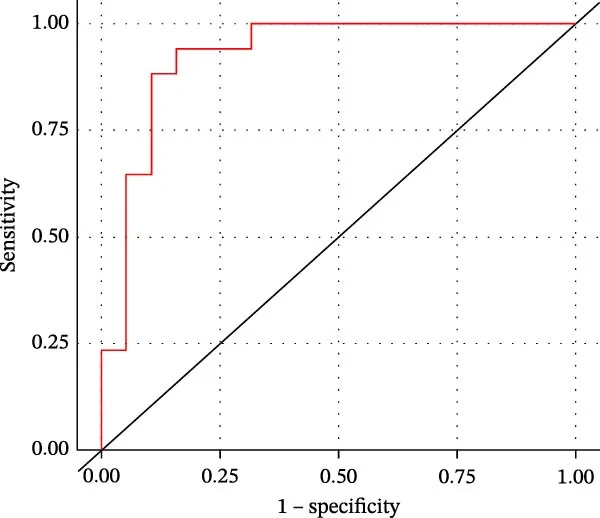
ROC curve for the global model.

The overall model was statistically significant (*p* < 0.001), with a McFadden’s *R* ^2^ of 0.494, indicating a good fit. The model demonstrated strong performance, with a sensitivity of 0.882, specificity of 0.895, precision of 0.889, and an AUC of 0.926 (Figure 1), reflecting excellent discriminative ability. The Youden index was 0.777, showing a strong balance between sensitivity and specificity. The results are summarized in Table 5.

**Table 5:** Backward stepwise logistic regression (BSLR) showing the best combination of variables for correctly classifying patients as UDD (*n* = 19) and BDD (*n* = 17).

Predictor	Estimation	Standard error	*Z*	*P*	Odds ratio	95% confidence interval	
						Inferior	Superior
Intercept	4.4995	9.65991	0.466	0.641	89.974	5.39e − 7	1.50e + 10
Rest per 24 h (%)	0.0688	0.08548	0.805	0.421	1.071	0.906	1.27
Sleep efficiency (%)	−0.0719	0.12513	−0.575	0.565	0.931	0.728	1.19
PSQI	−0.3926	0.27332	−1.436	0.151	0.675	0.395	1.15
ISI	−0.2784	0.18292	−1.522	0.128	0.757	0.529	1.08
N2% TST	0.1672	0.08549	1.955	0.051	1.182	1.000	1.40
L5 average	−5.95e−4	0.00141	−0.423	0.672	0.999	0.997	1.00

Deviance	AIC	*R* ^2^		General model			
				*X* ^2^		*ρ*	
25.2	39.2	0.494		24.6		<0.001	

**Specificity**	**Sensitivity**	**AUC**	**Youden index**	**PPV**		**NPV**	

0.895	0.882	0.926	0.777	0.883		0.895	

*Note*: Odds ratios (ORs) > 1 indicate association with BDD; OR < 1 indicate association with UDD.

Abbreviations: AIC, Akaike information criterion; AUC, area under the curve; ISI, insomnia severity index; NPV, negative predictive value; PPV, positive predictive value; PSQI, Pittsburgh sleep quality index; TST, total sleep time.

Additionally, the model showed strong reliability, with a PPV of 88.3% and a NPV of 89.5%, indicating high accuracy in identifying individuals with BDD and UDD, respectively.

However, when examining each variable individually, based on its *p*‐value, OR, and confidence interval, none was sufficient on its own to accurately classify patients into UDD and BDD.

## 4. Discussion

To our knowledge, this is the first study conducted in a routine care setting to compare patients diagnosed with UDD and BDD using such a broad range of subjective and objective sleep and circadian assessments. Our findings revealed distinct sleep and circadian profiles between the two groups. Patients with UDD reported poorer subjective sleep quality (PSQI) and more severe insomnia symptoms (ISI) and reduced SE as objectively measured by actigraphy compared to those with BDD. In contrast, patients with BDD displayed higher levels of rest over 24 h, with lower average activity during the least active 5‐h period (L5 average) and the 10 most active hours (M10 average) measured by actigraphy, a longer proportion of sleep spent in N2% TST and in total nonrapid eye movement (NREM), associated with low N3% TST measured in PSG.

Building on these differences, we identified a robust six‐factor model that distinguishes patients with UDD from BDD, explaining 49.4% of the variance. The key distinguishing factors include subjective measures (PSQI and ISI), actigraphic features (L5 average, rest time over 24 h%, and SE%), and polysomnographic indicators (N2% TST). The model demonstrated good discriminative power and strong predictive values. These results underscore the potential of combining subjective and objective sleep and circadian measures into a multidimensional predictive test to improve the differential diagnosis of UDD and BDD. Importantly, these features are also clinically meaningful, pointing to distinct sleep and circadian profiles between the two groups.

Notably, the multivariable model integrates several sleep and circadian markers, capturing different aspects of sleep regulation. Although no significant multicollinearity was observed, these variables likely reflect complementary dimensions of sleep functioning, including insomnia symptoms, global sleep quality, sleep continuity, sleep architecture, and circadian‐related motor activity, rather than a single redundant construct.

These findings may point toward a more “activated” depressive profile in UDD, with patients with UDD suffering from insomnia, weak sleep efficiency, and less time at rest over the 24‐h cycle compared to BDD patients. This global psychomotor activation could contribute to increased cognitive arousal, including ruminative processes, which have previously been associated with poorer subjective sleep quality in the literature [48–50]. Although our study did not directly assess rumination, this hypothesis would be consistent with previous neuroimaging studies. Indeed, several studies have reported increased connectivity within the default mode network (DMN) and reduced engagement of the salience network (SN) in patients with UDD compared to those with BDD [51, 52].

In contrast, in the model, patients with BDD exhibit a sleep profile with a predominance of N2 sleep % of TST compared to UDD, which is a stage of light slow sleep. Such a pattern may align with a hypoactive clinical profile, supported by actigraphic markers such as reduced L5 activity and extended resting periods across the 24‐h cycle [53]. This psychomotor slowing is consistent with historical clinical descriptions; Emil Kraepelin [54] previously characterized BDD as involving global psychomotor retardation, inhibition, and mental slowing. Moreover, this clinical concept has been supported by multiple studies indicating that psychomotor retardation could serve as a distinguishing marker of BDD [55–57]. Supporting this model, a neurophysiological perfusion study found increased resting‐state blood flow in motor‐related regions in patients diagnosed with BDD compared to that in UDD, suggesting a biological substrate for this motor and cognitive slowing [58].

Interestingly, the increase in total NREM duration found in the univariate analysis could be linked to the increase in N2% TST as N3% TST decreased. While N2 sleep contributes to memory consolidation via sleep spindles [59], it offers fewer restorative benefits than N3 sleep, which is more strongly associated with cognitive and physical recovery [60–62]. This imbalance may diminish the recuperative value of sleep, despite the longer subjective sleep duration reported by patients diagnosed with BDD, the trend toward hypersomnia (*p* = 0.065) and increased TST in PSG (UDD: 6 h 36 vs. BDD: 7 h 35; *p* = 0.060) that were found in univariate analysis. Rao et al. previously described a similar imbalance, characterized by increased N1 sleep and decreased deep sleep in adolescents experiencing a depressive episode who later converted to BD compared to those who remained within the UDD trajectory [63]. This could be consistent with clinical signs of daytime psychomotor slowing found in patients with BDD [64, 65].

Although all significant variables were included in the main model in line with our exploratory approach and given the limited statistical power of our model sample (*n* = 36), we performed an additional post hoc analysis to address the increased risk of false positives due to multiple comparisons. After applying a Benjamini–Hochberg correction, several variables remained significant at the univariate level (see Supporting Information, Tables S1–S4). These were then entered into a multiple regression model, in which the proportion of N2 sleep relative to TST (N2%) remained the unique significant predictor of BDD (OR = 1.193, 95% CI [1.032–1.380],  *p* = 0.017), (see Table S5). This suggests that N2%, in particular, may be the most robust marker for distinguishing the two groups in our sample. Most studies to date have not reported significant differences in N2 sleep between the two groups [14, 27], except a trend for increased spindles in BDD compared to UDD in a study by De Martelaer [12]. However, only a limited number of studies have specifically investigated sleep architecture in patients diagnosed with UDD and BDD and those that have often relied on smaller samples compared to ours. This novel finding is particularly interesting in that it may help illuminate a potential pathway underlying sleep and circadian disturbances in patients with BDD. An increase in N2 sleep might contribute to the fact that, despite a longer total sleep duration often observed in these patients, sleep remains nonrestorative. This, in turn, could help explain the cognitive impairments and psychomotor slowing frequently reported in this population [58, 64, 66].

Moreover, we found no differences in REM sleep between patients with BDD and UDD. Although some studies have reported such differences ([14]; “REM architecture changes in bipolar and unipolar depression,” 1979), REM alterations are common across several psychiatric disorders [67, 68] and therefore appear insufficient to reliably distinguish between UDD and BDD [13, 15, 27]. Regarding circadian rhythm markers, no significant differences were observed between groups in subjective and actigraphic measures, as we found in our previous studies [19]. However, this is not in line with a previous study [32], although their work was carried out in young adults.

However, this study has several limitations. One consideration involves medication: because mood stabilizers and antidepressants are first‐line treatments for BDD and UDD, respectively, their use is inherently collinear with the diagnostic status in this population, precluding their inclusion as covariates. Although this collinearity reflects real‐world clinical practice, a residual influence of medication cannot be excluded as antidepressants and mood stabilizers are known to influence both NREM and REM sleep, as well as circadian regulation [69–72].

Additionally, actigraphy and PSG could not be completed for all participants, mainly due to missed appointments, technical issues, or limited access to PSG early in the study. As these missing recordings were unrelated to clinical characteristics, this likely reflects real‐world operational conditions rather than a systematic selection bias.

While the model demonstrates strong predictive power, its explanatory capacity remains limited. The model explains only 49.5% of the variance (R^2^McF). This highlights the need to explore additional predictors to improve model fit and deepen our understanding of the underlying mechanisms distinguishing BDD and UDD. To achieve this, future studies should incorporate a broader range of variables and adopt more comprehensive diagnostic approaches.

Furthermore, while one of the strengths of these markers is that they are largely noninvasive and usable in clinical practice, PSG remains an examination that can be difficult to access. Ambulatory PSG may represent a promising avenue to facilitate future clinical applications.

In this context, a more integrative strategy combining data across multiple physiological dimensions becomes essential. The identified markers could be integrated into broader, multimodal approaches, such as AI‐based analytical tools, aligning with the ongoing transition toward advanced, data‐driven models of mental health [73, 74].

To the best of our knowledge, this is the first study to examine such a broad range of sleep variables in both unipolar and BDD. It reveals meaningful differences in sleep between the two groups. Importantly, it is also the first to propose a predictive model based on these sleep features, which shows promising classification performance.

## 5. Conclusion

In conclusion, this study supports the relevance of a multidimensional approach combining subjective complaints, actigraphic data, and polysomnographic measures to differentiate unipolar from BDD. Our predictive model showed strong classification performance, and our study identified N2% as a particularly robust marker. These results highlight meaningful differences in sleep profiles between the two disorders and suggest the potential of sleep‐related features as diagnostic tools.

## Funding

No funding was received for this manuscript.

## Disclosure

All edits were carefully reviewed and validated to ensure accuracy and clarity.

## Conflicts of Interest

The authors declare no conflicts of interest.

## Supporting Information

Additional supporting information can be found online in the Supporting Information section.

## Supporting information


**Supporting Information** Table S1: Sociodemographic and clinical characteristics of patients with bipolar depression (BDD, *n* = 43) and unipolar depression (UDD, *n* = 116) with Benjamini–Hochberg (BH) correction. Table S2: Comparison of patients who suffered from BDD (*n* = 43) and UDD (*n* = 116) regarding subjective sleep and circadian rhythm markers assessed with questionnaires with BH correction. Table S3: Sleep and circadian characteristics of patients with BDD (*n* = 42) and UDD (*n* = 93) assessed by actigraphy with BH correction. Table S4: Sleep and circadian characteristics of patients with BDD (*n* = 20) and UDD (*n* = 44) assessed by polysomnography with BH correction. Table S5: Logistic regression model after BH correction. Table S6: Collinearity diagnostics of the logistic regression variables.

## Data Availability

The data that support the findings of this study are available upon request from the corresponding author. The data are not publicly available due to privacy or ethical restrictions.
