# Supplementary material for: A Sleep and Circadian Biomarker‐Based Predictive Model for Differentiating Unipolar and Bipolar Depression
Source: Depress Anxiety. 2026 Sep 15;2026:5556144. doi: 10.1155/da/5556144 (PMC13579109; doi:10.1155/da/5556144)
Supplement: Supplementary file 1 [file DA-2026-5556144-s001.docx]

**Table S1:** Socio-demographic and clinical characteristics of patients with bipolar depression (BDD, n=43) and unipolar depression (UDD, n=116) with Benjamini-Hochberg (BH) correction.

| **Variables** | | **Groups mean (SD) or n (%)** | | **Comparison UDD vs BDD** | | |
| --- | --- | --- | --- | --- | --- | --- |
|  | | **UDD**  **(n=116)** | **BDD**  **(n=43)** | **X^2^, Mann- Whitney U, T-student, Welch** | ***ρ*** | ***ρ**** |
| **Age** (years) | | 39.50 (±13.32) | 43.49(±13.28) | -1,677 | 0.098 | 0.2246 |
| **Gender distribution** | Female | 73 (62.9%) | 25 (58.1%) | 0.305 | 0.581 | 0.6972 |
|  | Male | 43 (37.1%) | 18 (41.9%) |  |  |  |
| **Age of onset (**years) | | 25.1 (±10.6) | 23.0 (±9.62) | 1572 | 0.430 | 0.5733 |
| **Personal Hx of Trauma** | Yes | 61 (53%) | 18 (42.9%) | 1.28 | 0.258 | 0.3870 |
|  | No | 54 (47%) | 24 (57.1%) |  |  |  |
| **BMI** (kg/m^2^) | | 26.7 (±7.19) | 25.1 (±4.27) | 1.68 | 0.095 | 0.2246 |
| **Rating scales** | | | | | | |
| MADRS | | 25.03 (±7.75) | 24.32 (±7.32) | 1897 | 0.970 | 0.9700 |
| GAD-7 | | 12.62 (±0.476) | 12.76 (±0.785) | 2228 | 0.801 | 0.8738 |
| **Sleep symptoms of the current major depressive episode** | | | | | | |
| Insomnia | Yes | 98 (±84.5) | 31 (±73.8) | 2.34 | 0.126 | 0.2246 |
|  | No | 18 (±15.5) | 11 (±26.2) |  |  |  |
| Hypersomnia | Yes | 22 (±19.3) | 14 (±33.3) | 3.41 | 0.065 | 0.2246 |
|  | No | 92 (±80.7) | 28 (±66.7) |  |  |  |

*Hx: history, BMI: Body mass index, GAD-7: Generalized Anxiety Disorder-7, MADRS: Montgomery- Åsberg Depression Rating Scale, ρ*: post-hoc analysis, Benjamin-Hochberg correction*

**Table S2:** Comparison of patients suffered from bipolar depression (BDD= 43) or a Unipolar depression (UDD= 116) regarding subjective sleep and circadian rhythms markers assessed with questionnaires with BH correction.

| **Variables** | | **Groups mean (SD)**  **or n (%)** | | **Comparison UDD vs BDD** | | |
| --- | --- | --- | --- | --- | --- | --- |
|  | | **UDD**  **(n=116)** | **BDD**  **(n=43)** | **T-student, Mann-Whitney U** | ***ρ*** | ***ρ**** |
| **ISI** | | 18.98 (±4.87) | 16.60 (±5.78) | 1662 | **0.026** | **0.0446** |
| **PSQI** | | 12.83 (±3.83) | 10.61 (±3.51) | 3.2440 | **0.001** | **0.0040** |
|  | Subjective quality | 2.36 (±0.68) | 1.93 (±0.95) | 1800 | **0.010** | **0.0300** |
|  | Sleep latency | 2.02 (±0.94) | 1.62 (±0.96) | 1811 | **0.018** | **0.0360** |
|  | Sleep duration | 1.11 (±1.20) | 0.64  (±0.98) | 2.4987 | **0.014** | **0.0336** |
|  | Sleep efficiency | 1.56 (±1.20) | 0.83 (±1.08) | 1551 | **<0.001** | **0.0040** |
|  | Sleep disturbance | 1.91 (±0.67) | 1.50 (±0.60) | 1635 | **<0.001** | **0.0040** |
|  | Sleep promoting medication | 1.98 (±1.30) | 2.38 (±1.13) | -1.8809 | 0.063 | 0.0945 |
|  | Daytime dysfunction | 1.88 (±0.78) | 1.73 (±0.78) | 2096 | 0.333 | 0.4044 |
| **ESS** | | 9.17 (±5.32) | 8.65 (±5.35) | 2296 | 0.541 | 0.5902 |
| **H&O** | | 46.24 (±11.17) | 45.77 (±13.51) | 0.2128 | 0.832 | 0.8320 |
| **HSI** | | 18.43 (±5.33) | 17.38 (±5.86) | 0.9642 | 0.337 | 0.4044 |

*ISI: Insomnia Severity Index, PSQI: Pittsburg Sleep Quality Index, ESS: Epworth Sleepiness Scale, H&O: Horne & Östberg, HSI: Hypersomnia severity index , ρ*: post-hoc analysis, Benjamin-Hochberg correction*

**Table S3:** Sleep and circadian characteristic of patients with bipolar depression (BDD) (n=42) or a unipolar depression (UD) (n=93) assessed by actigraphy with BH correction.

| **Variables** | **Groups mean (SD) or n (%)** | | **Comparison UDD vs BDD** | | |
| --- | --- | --- | --- | --- | --- |
|  | **UDD**  **(n=93)** | **BDD**  **(n=42)** | **T- student, Mann-Whitney U, Welch** | ***ρ*** | ***ρ**** |
| **TST**  (hh:mm:ss) | **7:05:05 (±1:12:12)** | **7:27:57 (±1:26:56)** | -1.4885 | 0.141 | 0.2585 |
| **WASO**  (hh:mm:ss) | **1:23:27 (±0:35:27)** | **1:10:51 (±0:30:25)** | 1595 | 0.90 | 0.9710 |
| **Sleep efficiency**  (SE) (%) | 80.703  (±7.06) | 83.619 (±6.807) | -2.2469 | **0.026** | 0.0715 |
| **SOL**  (hh:mm:ss) | **0:13:36 (±0:12:18)** | **0:13:30 (±0:23:42)** | 0.0359 | 0.971 | 0.9710 |
| **Rest per 24h**  (%) | 44.147  (±11.079) | 49.102 (±11.052) | -2.4073 | **0.017** | 0.0715 |
| **L5 onset**  (hh :mm :ss) | **3:25:08 (±5:24:50)** | **2:47:08 (±5:35:53)** | 1536 | 0.076 | 0.1672 |
| **L5 average** | 1105.478 (±848.402) | 784.524  (±822.565) | 1295 | **0.004** | **0.0440** |
| **M10 onset** (hh :mm :ss) | **10:17:20 (±2:17:21)** | **10:10:00 (±1:48:24)** | 1791 | 0.626 | 0.7651 |
| **M10 average** | 12308.833 (±5006.364) | 10028.095  (±4495.531) | 1427 | **0.024** | 0.0715 |
| **Interdaily stability** (IS) | 0.375  (±0.119) | 0.386  (±0.129) | -0.4921 | 0.623 | 0.7651 |
| **Intradaily variability** (IV) | 0.871  (±0.193) | 0.906  (±0.237) | 1765.5 | 0.545 | 0.7651 |

*TST: total night sleep time, WASO: wake after sleep onset, SOL: sleep onset latency; M10 onset: most 10 start hour onset, M10 average: average activity of the 10 most actives hours, L5 onset: low 5 start hour onset, L5 average: average activity of the 5 most actives hours, ρ*: post-hoc analysis, Benjamin-Hochberg correction*

**Table S4:** Sleep and circadian characteristics of patients with BDD (n=20) and UDD (n=44) assessed by polysomnography with BH correction.

| **Variables** | **Groups mean (SD) or n (%)** | | **Comparison UDD vs BDD** | | |
| --- | --- | --- | --- | --- | --- |
|  | **UDD**  **(n=44)** | **BDD**  **(n=20)** | **T- student, Mann-Whitney U, Welch** | ***ρ*** | ***ρ**** |
| **TST**  (hh:mm:ss) | **6:36:06**  **(±1:34:00)** | **7:35 :30 (±1:21:36)** | -1.920 | 0.060 | 0.1500 |
| **WASO**  (hh:mm:ss) | **0:52:43**  **(±1:03:26)** | **0:59:00 (±1:02:44)** | 91 | 0.647 | 0.7189 |
| **SE** (%) | 79.1  (±16.2) | 85.3  (±14.6) | 133 | 0.582 | 0.7189 |
| **SOL**  (hh:mm:ss) | 00 :33 :20  (±00 :48 :28) | 00 :25 :02  (±00 :43 :42) | 158 | 0.937 | 0.9370 |
| **N1 TST (%)** | 8.718  (±7.725) | 7.585  (±6.319) | 0.574 | 0.568 | 0.7189 |
| **N2 TST (%)** | 48.298  (±10.966) | 56.560  (±9.151) | -2.934 | **0.005** | **0.0500** |
| **N3 TST (%)** | 25.641  (±11.644) | 19.380  (±6.673) | 2.237 | **0.029** | 0.0967 |
| **Duration of NREM** (hh:mm:ss) | 04 :59 :16  (±01 :16 :32) | 05 :49 :38  (±01 :04 :44) | -2.554 | **0.013** | 0.0650 |
| **REM TST (%)** | 17.436  (±8.523) | 16.375  (±8.198) | 0.467 | 0.642 | 0.7189 |
| **REM latency (min)** | 140.82  (±83.45) | 208.98  (±167.54 ) | -1.547 | 0.139 | 0.2780 |

*TST: total sleep time, WASO: Wake after sleep onset, SE: sleep efficiency, SOL: sleep onset latency, NREM: Non-Rapid eyes movement, REM: Rapid eyes movement, ρ*: post-hoc analysis, Benjamin-Hochberg correction.*

**Table S5**: Logistic regression model after BH correction.

|  | | | | | | **95% confidence interval** | |
| --- | --- | --- | --- | --- | --- | --- | --- |
| **Predictor** | **Estimation** | **Standard Error** | **Z** | **P** | **Odds ratio** | **Inferior** | **Superior** |
| Intercept | 0.561 | 4.09606 | 0.137 | 0.891 | 1.753 | 5.72e-4 | 5374.03 |
| ISI | -0.313 | 0.18130 | -1.725 | 0.085 | 0.731 | 0.513 | 1.04 |
| **N2 %TST** | **0.177** | **0.07401** | **2.386** | **0.017** | **1.193** | **1.032** | **1.38** |
| PSQI | -0.277 | 0.21785 | -1.271 | 0.204 | 0.758 | 0.495 | 1.16 |
| L5 Average | -6.11e−4 | 0.00102 | -0.597 | 0.551 | 0.999 | 0.997 | 1.00 |

*AIC: Akaike Information Criterion****,*** *AUC: Area under the curve, ISI: insomnia severity index, TST: total sleep time, NPV: Negative Predictive Value, PPV: Positive Predictive Value, PSQI: Pittsburg Sleep Quality Index*

*Note: Odds ratios (OR) > 1 indicate association with BDD; OR < 1 indicate association with UDD.*

**Table S6 :** Collinearity diagnostics of the logistic regression variables

| **Variables** | **VIF** | **Tolerance** |
| --- | --- | --- |
| Rest per 24h % | 2.65 | 0.377 |
| SE % | 2.82 | 0.355 |
| PSQI | 2.12 | 0.472 |
| ISI | 1.95 | 0.514 |
| N2 % TST | 1.75 | 0.572 |
| L5 average | 1.82 | 0.550 |

*ISI: Insomnia Severity Index, SE: Sleep efficiency, PSQI: Pittsburg Sleep Quality Index.*
